# A Prospective Study of Mid-Trimester MCP-1 Levels as a Predictor of Preterm Delivery

**DOI:** 10.3390/medicines10010007

**Published:** 2022-12-30

**Authors:** Mirjana A. Bogavac, Dejan D. Ćelić, Tamara M. Perić

**Affiliations:** 1 Clinical Center of Vojvodina, Department of Obstetrics and Gynecology, Faculty of Medicine, University of Novi Sad, Hajduk Veljkova 3, 21000 Novi Sad, Serbia; 2 Clinical Center of Vojvodina, Department of Nephrology and Clinical Immunology, Faculty of Medicine, University of Novi Sad, Hajduk Veljkova 3, 21000 Novi Sad, Serbia; 3 Faculty of Medicine, University of Novi Sad, Hajduk Veljkova 3, 21000 Novi Sad, Serbia; 4 PHI Hospital “Sveti Vračevi”, Srpske Vojske 53, 76300 Bijeljina, Bosnia and Herzegovina

**Keywords:** MCP-1, preterm delivery, prediction markers

## Abstract

**Background**: The prevention of preterm delivery (PTD) represents one of the major topics in modern obstetrics. The aim was to design a prospective study and investigate if mid-trimester serum and amniotic fluid levels of MCP-1 could predict the occurence of spontaneous PTD. **Methods:** The study involved 198 women who underwent genetic amniocentesis and blood sampling in the middle of their trimester. After applying the criteria for inclusion in the study, there were 16 respondents in the study group, and 38 respondents in the control group. Level of MCP-1 in amniotic fluid and serum was measured with commercially available enzyme-linked immunosorbent assays (ELISA) and statistical analysis was conducted. **Results:** There was no statistically significant difference in serum or amniotic fluid MCP1 levels between PTD and the control groups. **Conclusion:** The results suggest that MCP-1 is probably not the most relevant marker for predicting PTD. This study provides new normative data for MCP-1 levels in amniotic fluid and maternal sera and is a valuable tool for future diagnostic and comparative studies.

## 1. Introduction

Preterm delivery (PTD) is defined as giving birth before the 37th week of gestation. PTD is one of the biggest problems in modern obstetrics. Approximately 8% of pregnancies in Serbia end with PTD and this is a significant cause of perinatal morbidity and mortality [1]. There are numerous difficulties in preventing PTD. PTD is the common consequence of numerous pregnancy complications involving the cervix, fetus, fetal membranes, placenta, and myometrium [2]. Medical efforts have focused on mitigating the consequences of preterm birth rather than preventing it. Prevention is what is needed [3], and current research is focused on achieving this goal. 

Spontaneous labor at term is associated with infiltration of inflammatory cells in the cervix, myometrium, chorioamniotic membranes, and the amniotic cavity of women in labor [2,4]. This is associated with the increased production of proinflammatory cytokines and chemokines, including monocyte chemoattractant protein-1 (MCP-1). MCP-1 serves not only as a specific monocyte chemotactic agent, but was also initially characterized as a product of activated monocytes. It has been identified in a variety of cell types, and MCP-1 expression has been observed in tissues such as decidua, endometrium, chorion, and placenta. MCP-1 plays an integral role in the control of a normal pregnancy, as well as host response to intrauterine infection, and potential PTD. Studies have shown that a significant percentage of premature births are associated with increased activity of immune cells in the uterus [5,6,7]. At present, it is accepted that proinflammatory cytokines play a central role in the mechanisms of term and inflammation/infection-induced preterm delivery. Although evolution has shaped the normal physiology of human childbirth, premature birth can be the result of either premature activation of these normal mechanisms for preterm birth or pathological influences that trigger childbirth in different ways. It is now an accepted fact that intrauterine infection causes a significant percentage of spontaneous PTDs [6]. Bacteria can rise from the lower genital tract before or during pregnancy, infect membranes and initiate an inflammatory response that culminates in PTD or preterm premature rupture of membranes (PPROM) [2,8]. However, in many cases, the infection remains subclinical. Occult (hidden) infection of the normally sterile amniotic cavity can activate a cascade of inflammatory mediators that stimulate the synthesis and release of prostaglandins, which ultimately leads to uterine contractions and irreversible changes in the cervix [9,10].

In the moment of embryo implantation, a successful pregnancy requires two main elements: a quality embryo and endometrial receptivity. Cytokines released at the feto–maternal interface play a central role in fetal survival, not only by affecting the maternal immune system, but also by regulating angiogenesis and vascular development [11,12]. Endometrial remodeling in pregnancy involves the local accumulation of leukocytes, including natural killer cells and macrophages [13]. Blastocysts secrete various cytokines, chemokines, and other immune factors, while human embryos secrete an abundance of cytokines and chemokines, and one of them is monocyte chemotactic protein-1 (MCP-1). MCP-1 (CCL-2) is produced by different types of cells, including endothelial cells, fibroblasts, monocytes, lymphocytes, and smooth muscle cells [14]. In the reproductive system, MCP-1 is produced by trophoblasts, decidual, endometrial, and myometrial cells and has been suggested to contribute to the onset or propagation of normal or premature labor. Several different pieces of evidence using in vivo animal models suggest that MCP is an important factor in inflammatory processes [15]. Studies on MCP—1/JE deficient mice include roles in angiogenesis and the Th1 polarization of naive T cells [16]. More than one study showed that MCP-1 levels in endometrial secretions are associated with implantation [17,18]. The expression of MCP-1 was found to be significantly increased during childbirth compared to the resting human endometrium [14]. High levels of circulating chemokines have been observed in many pathological processes and can serve as early indicators of unfavorable pregnancy outcomes [19,20]. Levels of MCP-1 are significantly increased at birth in amniotic fluid and cervical secretions in women experiencing both term and PTD. The mechanisms that regulate myometrial MCP-1 expression are unknown. 

Hence, the goal of this study was to investigate if mid-trimester serum and amniotic fluid levels of MCP-1 could predict the occurrence of spontaneous PTD. We hypothesized that MCP-1 levels would be higher in patients with preterm delivery. 

## 2. Materials and Methods

This was a prospective study designed to examine the relationship between mid-trimester serum MCP-1 levels and amniotic fluid and the occurrence of spontaneous preterm birth. The study is approved by the Ethics Committee of the Clinical center of Vojvodina and the Faculty of Medicine University of Novi Sad (Approval Code: 00-05/508). All participants signed the informed consent form, which is in accordance with the criteria of the Helsinki Declaration. All amniocenteses were performed between the 16th and 19th weeks of gestation. Each amniocentesis was preceded by a detailed ultrasound scan with a 3.75 MHz curvilinear transducer to assess fetal anatomy and to determine the location of the placenta. The gestational age was assessed either by the last menstrual period or by an early ultrasound scan if there was a discrepancy of more than a week. Inclusion criteria included singleton pregnancy, normal pregnancy course prior to the procedure, maternal age > 18 years, intact fetal membranes, no signs of preterm labor or cervical dilatation at the time of amniocentesis, and undiagnosed fetal anomalies with an anatomy ultrasound scan. We excluded cases with abnormal fetal karyotype, major fetal anomalies, and significant medical or obstetric complications such as preeclampsia, fetal growth restriction, gestational diabetes, polyhydramnios or oligohydramnios, or placenta previa leading to iatrogenic preterm delivery from the analysis.

Our subjects were 198 women who underwent genetic amniocentesis and blood sampling in the middle of their trimester. Among them, 5 fetuses were born <37 weeks due to indications for the fetus or mother. Abnormal ultrasounds in the second trimester were found in 3 women. Chromosomal abnormalities were found in 2 women. All of these cases underwent pregnancy termination. Four women were lost for follow-up and were therefore excluded from the analysis. The remaining 184 women met the inclusion criteria. 

Of those 184 subjects, 16 of them (8.7%) had PTD and represented the study group. The remaining 168 subjects had a term delivery. Thirty-eight women were randomly selected using the random selection method to represent the control group.

The study group included all women who had PTD (<37 weeks of gestation). The control group consisted of the women who were matched by maternal age, parity, and indication for amniocentesis, who also underwent amniocentesis and blood sampling in the same time period and who had term labor.

Samples: Blood samples were taken from the antecubital vein in EDTA anticoagulant tubes and then centrifuged at room temperature with 3000× *g* for 10 min. Serum aliquots were stored at −20 °C until analysis.

Amniotic fluid samples were collected during genetic amniocentesis in polypropylene tubes. After centrifugation at 200× *g* for 10 min, supernatant was collected and stored at −20 °C within 1 h of the procedure, until analysis.

Cytokines: Levels of MCP-1 in amniotic fluid and serum were measured with commercially available enzyme-linked immunosorbent assays (ELISA) (R&D Systems, Inc. Minneapolis, USA). The ELISA was validated for amniotic fluid, and the samples were measured in duplicate with a microplate reader (Beckman Coulter). Sensitivity of the assay was 5.0 pg/mL, and intra-assay and inter-assay coefficients of variation were 6.8% and 7.9%, respectively.

Statistical analysis: A Mann–Whitney test was used to compare continuous variables within the two groups. Chi-square and Fisher’s exact tests were performed for the comparison of proportions. Analyses were conducted using Statistical Package for Social Sciences IBM SPSS Statistics for Windows, Version 23 (IBM Corp., Armonk, NY, USA). For all statistical analyses, *p* < 0.05 was considered statistically significant.

## 3. Results

Table 1 presents the demographic and clinical characteristics of the two investigated groups. There are no statistically significant differences between groups in these parameters: maternal age, number of previous pregnancies, gestational age at sampling, and number of previous preterm births. Serum MCP-1 levels were four times lower compared to amniotic fluid in women with PTD and ten times lower in women without PTD. There was no statistically significant difference in serum or amniotic fluid level MCP-1 between the groups of patients.

## 4. Discussion

Several studies on MCP-1 conducted in the last ten years emphasize the importance of this cytokine for preterm labor and delivery. Findings of elevated levels of MCP-1 in the cervix of women and significantly elevated MCP-1 levels in the amniotic fluid of women in premature labor with or without evidence of infection were reported [5,21]. In a study by Kim et al. (2020), significantly higher levels of seven investigated proteins were confirmed, including MCP-1, in the amnionic fluid and cervicovaginal fluid (none in the plasma) found in the women with intra-amniotic infection and/or inflammation (and indirectly, PTD) than in those without intra-amniotic infection and/or inflammation [5]. MCP-1 is chemotactic for monocytes, macrophages, lymphocytes, and basophils. In a study by DeRen Huang et al. (2001), the influence of MCP-1 in the inflammatory process was investigated and the authors reported that MCP-1 appears to have intrinsic immune functions [22]. However, the contributions of MCP-1 to the inflammatory response seems to be pleiotropic. Normal amniotic fluid concentrations for MCP-1 were found to be significantly elevated over maternal serum concentrations in the matched pairs (women with and without PTD) (*p* < 0.05). This elevation was reported by Chow et al. (2008) and suggests that amniotic fluid may be more representative of the fetal cytokine profile than cytokine analysis on antenatal sera as it represents predominantly fetal urinary and respiratory secretions [23]. In this study, elevated concentrations of MCP-1 in the serum of the mother and in the amniotic fluid of women who gave birth before term were reported. This is in agreement with previous reports, where higher levels of this cytokine in women with PTD, compared to women without PTD, is reported [5,24,25]. In contrast, in our study, no statistically significant differences in serum or amniotic fluid level MCP-1 between term and premature birth were found. Based on the presented results, the working hypothesis that MCP-1 levels would be higher in patients with preterm delivery was rejected. Our results showed that MCP-1 is also constitutively present in human amniotic fluid during the second trimester, suggesting that MCP-1 plays a role in the early development of a normal pregnancy, which is in agreement with the results of the study of Esplin et al. (2003). The authors concluded that amniotic fluid levels of MCP-1 increase during spontaneous labor at term [26].

In a study by Zhou et al. (2019), it was reported that expression levels of serum MCP-1 were elevated in preterm parturient women, particularly in those with infective premature delivery, which suggests that MCP-1 can be used as predictors of infectious preterm birth [27].

The major finding of the study by Jacobsson et al. (2003) was that amnion fluid MCP-1 is associated with microbial invasion and inflammation of the amniotic cavity in women with pPROM, as well as PTD [28].

Our study had several limitations. The main limitations are the relatively small number of cases included in the study. Therefore, further large-scale prospective cohort studies are required to confirm our findings in other study populations. The second limitation is the longevity of the period between sampling the material (maternal serum and amniotic fluid) and the occurrence of PTD; this period was about 14 weeks.

## 5. Conclusions

In this study we conducted an investigation to determine if mid-trimester serum and amniotic fluid levels of MCP-1 could predict the occurrence of spontaneous PTD. Based on results obtained, this study suggests that MCP-1 is probably not the most relevant marker for predicting PTD, but further trials on a larger sample are necessary to confirm the findings. 

## Figures and Tables

**Table 1:** Demographic and clinical characteristics of women according to the gestational age of delivery.

	Preterm Delivery (PTD)		*p* Value
	Yes (N = 16)	No (N = 38)	
Maternal age, mean ± SD	36.2 ± 3.2	35.7 ± 3.6	0.263
Previous PTDs; N (%)	2 (8)	7 (14)	0.221
MCP-1 in serum, median (interquartile range) (pg/mL)	217 (66)	75 (50)	0.669
MCP-1 in amniotic fluid, median (interquartile range) (pg/mL)	809 (172)	759 (126)	0.933

Legend: SD—standard deviation; PTD—preterm delivery; MCP-1—monocyte chemoattractant protein-1.

## Data Availability

Not applicable.
